# Air crescent sign in a case of aspergilloma

**DOI:** 10.11604/pamj.2024.48.71.43019

**Published:** 2024-06-27

**Authors:** Aishwarya Kishor Kedar, Babaji Ghewade

**Affiliations:** 1 Department of Respiratory Medicine, Datta Meghe Institute of Higher Education and Research, Wardha, Maharashtra, India

**Keywords:** Air crescent sign, Monod sign, *Aspergillus fumigatus*, aspergilloma

## Image in medicine

A sixty-year-old female presented with complaints of fever, cough with expectoration, and breathlessness on exertion for 20 days. She had a history of pulmonary tuberculosis 6 years back for which she had fully completed the course. She was vitally stable. Her systemic examination revealed bronchial breath sounds in the left suprascapular area. Chest radiography was done for the patient which revealed fibrotic changes in bilateral lung fields. Her sputum examination came out to be negative for acid-fast bacilli and nucleic acid amplification test. High-resolution computed tomography of the lungs was further carried out and revealed a cavity in the left upper lobe with a rounded mass in it. The mass was separated from the wall of the cavity by a crescent-shaped airspace (Air crescent sign). There was a change in the position of the mass within the cavity after the change in the position of the patient (Monod sign). The patient was further investigated for serum IgG antibody for *Aspergillus fumigatus* and it came out to be positive thus confirming the diagnosis of fungal ball or aspergilloma. The patient was discharged after antifungal therapy and symptomatic treatment and advised for regular follow-up.

**Figure 1:**
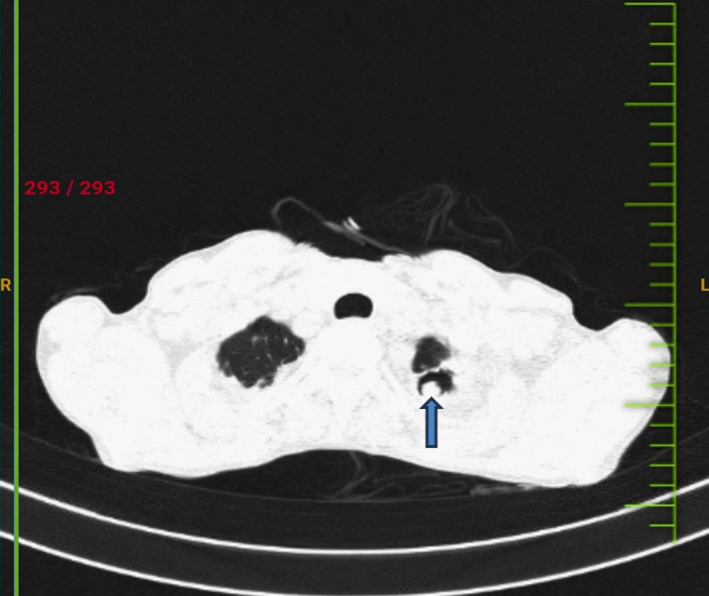
axial section of computed tomography of lung showing a cavity in left upper lobe (blue arrow) with a rounded mass in it with a surrounding crescent shaped air shadow (the Air crescent sign)

